# Schwannoma of the Brachial Plexus: A Rare Case Report

**DOI:** 10.22038/ijorl.2020.40635.2330

**Published:** 2020-07

**Authors:** Shruti Ranjan, Nikhil Arora, Deepika Sethi, Daljeet Kaur, Gyanesh Sethi

**Affiliations:** 1 *Department of ENT & Head and Neck Surgery* *Dr. Baba Saheb Ambedkar Medical College and Hospital *

**Keywords:** Brachial plexus, Neurofibroma, Schwannomas

## Abstract

**Introduction:**

Brachial plexus schwannomas are extremely rare tumours of the head and neck region accounting for less than 5 % overall. Due to its rarity and anatomic complexity of the brachial plexus, schwannomas in this region present a diagnostic and surgical challenge to the surgeon.

**Case Report:**

We present a case of a 56-year-old female who presented with a slow growing right sided neck swelling associated with pain and tingling in the distal end of the right forearm. According to FNAC, imaging studies results, a diagnosis of benign neurogenic tumour possibly schwannoma was made. After taking proper consent patient underwent surgical excision of the tumour. Postoperatively, patient developed numbness and tingling in right arm and stiffness at elbow joint, which is showing improvement after regular physiotherapy sessions.

**Conclusion:**

Although brachial plexus schwannomas are extremely rare head and neck tumours they should be kept as a differential diagnosis in patients presenting with supraclavicular neck swellings. These are potentially curable lesions. As such, detailed history and examination together with imaging studies is important in establishing a preoperative diagnosis for proper management.

## Introduction

Brachial plexus schwannomas are extremely rare tumours of the head and neck region. Schwannomas are benign tumours arising from Schwann cells of peripheral nerves. Up to half of these occur in the head and neck region, only 5% of which arise from the brachial plexus. These tumours usually present as a painless palpable mass in the neck. We hereby present a case who presented to our out-patient department (OPD) with a slow growing right sided neck swelling which was eventually diagnosed as schwannoma of the brachial plexus. We also review such cases previously reported in literature. Our case highlights that due to the rarity and anatomic complexity of the brachial plexus, schwannomas in this region present a diagnostic and surgical challenge to the surgeon.

## Case Report 

**Fig 1:**
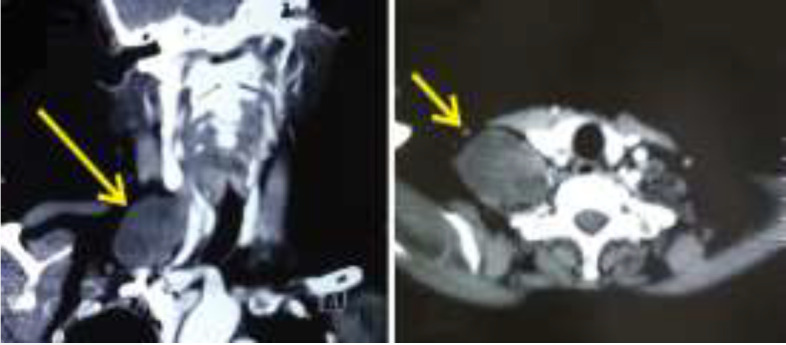
Cect Neck: Well-defined heterogeneously enhancing solid cystic lesion in right supraclavicular region medially extending into and causing widening of right C6-C7 neural foramen

A 56-year-old female presented to OPD with complaints of right sided supraclavicular neck swelling for last 2 years and associated pain and tingling in the right arm for past 1 year. There was no history of fever, trauma, systemic illness. On examination, a 5X4 cm spherical swelling was palpable in the right supraclavicular area which was non tender, not fixed to the overlying skin and it was more mobile in the horizontal direction than in the vertical direction. On neurological examination, muscle power in all muscles of the upper limb was found to be 5/5 with no signs of wasting and sensations were intact. Tinel sign was negative. Decision to take a FNAC of the swelling was taken which revealed spindle shaped cells and schwann cells which was suggestive of schwannoma. Furthermore, CECT neck was done which showed a heterogeneously enhancing solid cystic lesion in the right supraclavicular region medially extending into and causing widening of right C6-C7 neural foramen (Fig.1).

**Fig 2:**
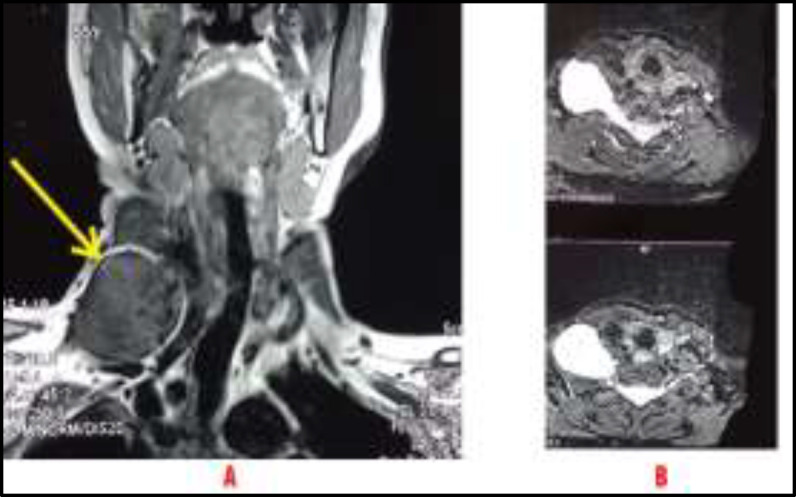
MRI Neck And Chest: Well defined solid cystic lesion in right supraclavicular region: (A) Isointense on T1WI; (B) Heterogenous enhancement seen on post contrast scan

MRI neck and chest were also ordered which showed a well-defined solid cystic lesion in right supraclavicular region which was isointense on T1WI and heterogenous enhancement was seen on post contrast scan which confirmed our diagnosis of Schwannoma most probably arising from the brachial plexus (Fig.2). 

**Fig 3:**
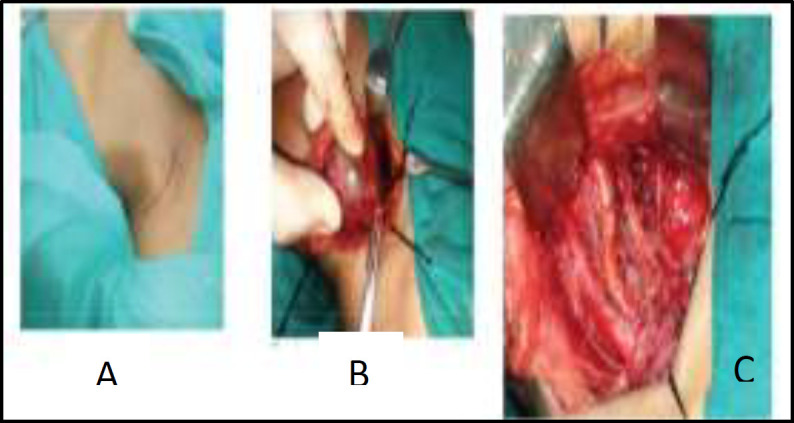
(A) Horizontal incision over the swelling; (B) Trunks of the Brachial Plexus passing around the tumour; (C) Brachial Plexus after tumour removal

After a thorough evaluation of the case, and taking proper consent from the patient after explaining all possible complications, the decision of undertaking excision of the tumour under general anaesthesia was made. A horizontal incision was given over the swelling and tumour was exposed. Trunks of the brachial plexus were seen passing around the tumour (Fig.3). 

The tumour was carefully excised without causing any damage to the trunk and sent for histopathological examination.

**Fig 4:**
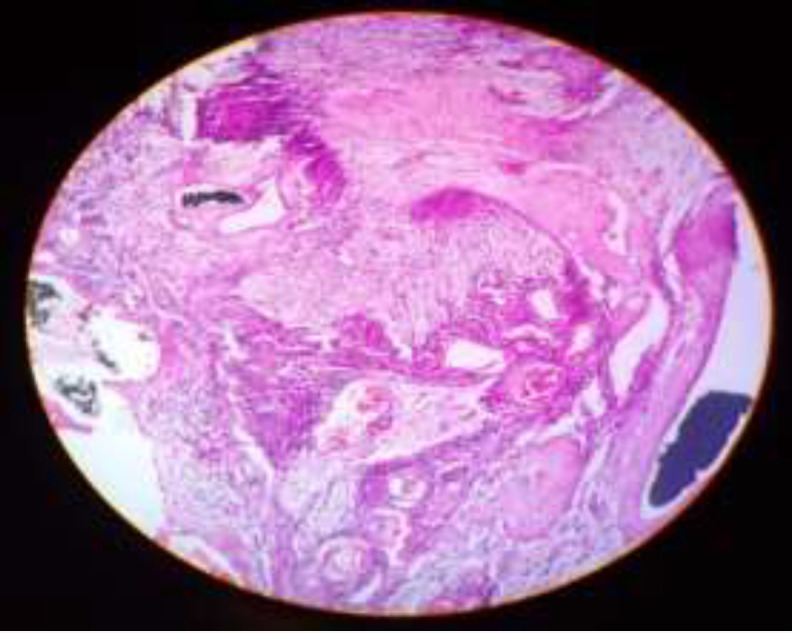
Characteristic cellular pattern of alternating regions containing compact spindle cells called Antoni type A areas and more loosely arranged, hypocellular zones called Antoni type B areas

The histopathology of the tumour specimen showed a characteristic cellular pattern of alternating regions containing compact spindle cells called Antoni type A areas and more loosely arranged, hypocellular zones called Antoni type B areas which was consistent with the diagnosis of Schwannoma (Fig.4).

Hence, a final diagnosis of schwannoma of the Brachial plexus was made. In the postoperative period, numbness and tingling in the right arm along with stiffness at the elbow joint was noticed. Patient is being regularly followed up in the last 6 months and has shown improvement in stiffness, tingling and numbness with regular physiotherapy.

## Discussion

Schwannomas or neurilemmomas are benign, well encapsulated, usually solitary tumours arising from the schwann cells of the peripheral nerves. Up to half of these arise in the head and neck region from cranial nerves, sympathetic chain, cervical nerve roots and brachial plexus. Brachial plexus tumours are extremely rare entities and form only 5% of all the upper limb tumours. The most common brachial plexus tumours are schwannomas and neurofibromas. The most common site of the brachial plexus tumours is head and neck. Head and neck region comprise of 25-45% of all the schwannomas of which only 5% arise from the brachial plexus (1).

Schwannomas usually present as slow growing painless tumours. However, they may also be accompanied with symptoms secondary to nerve compression. Proper evaluation including detailed history and examination together with the imaging studies help in establishing preoperative diagnosis and avoiding undue misdiagnosis. MRI is usually the study of choice and schwannomas typically present as a well circumscribed mass having heterogenous and bright areas on T2WI with post contrast enhancement. CECT may further aid in diagnosis where most schwannomas are iso-dense to brain parenchyma.

To highlight the importance of imaging studies in aiding the diagnosis of schwannoma, Ryu KH et al reported about a case who presented with a palpable neck swelling for one year. A differential of metastatic cervical lymphadenopathy was made. However, CT and MRI along with US guided core needle biopsy proved it to be a schwannoma (2).

Rashid M et al also reported two cases of brachial plexus schwannoma involving C7 root presenting as supraclavicular neck swelling. One of their cases had previously been misdiagnosed as enlarged supraclavicular lymph node and an attempt of excisional biopsy from the same swelling had resulted in iatrogenic injury. Thus, they highlighted the importance of establishing a proper diagnosis of the lesion with the help of FNAC and imaging studies to avoid any misdiagnosis and hence mismanagement. (3)

Our case also presented in her fifth decade with a slow growing mass in her neck region. After a thorough history and examination, we decided to perform FNAC of the swelling, which was suggestive of schwannoma. MRI and CECT neck were also carried out to further help in aiding the diagnosis. Thus, FNAC and imaging studies are extremely important in preventing misdiagnosis and hence mismanagement which can have untoward complications. 

Surgical resection with preservation of the surrounding nerves is typically the treatment of choice as schwannomas are radioresistant. Neurological dysfunction, pain, rapidly growing tumours with suspicion of malignancy are usually the indications for surgical resection. Grossly, these tumours are round, oval or plexiform and can be yellow, grey, pink or tan. Histopatholgy, helps in confirming the diagnosis showing typical Antoni type A and B patterns with rows of nuclear palisading (verocay bodies).

Unlike neurofibromas which are unencapsulated and usually envelop the nerve fascicles, schwannomas are generally well encapsulated and displace the nerve fascicles. However, while schwannomas can be enucleated from the nerve, post-operative neurological deficit is still common and as such preoperative counselling of patients regarding the same is extremely important in the overall management.

Kumar A et al, in their case report, highlighted the importance of preoperative counselling regarding neurological deficit following surgical resection of brachial plexus schwannoma. They reported of a case in which the patient presented with an axillary swelling. The tumour was resected via an axillary approach and postoperatively the patient developed loss of sensation over index finger and thumb. (4)

Vishwanathan N et al also reported of a case of brachial plexus schwannoma, in which intra-operatively, the tumour was found to be splayed into very thin effaced sheet of neural tissue. Despite all efforts made microscopically, the upper trunk C5-C6 nerves had to be severed resulting in post-operative neurological deficit. The surgeons then planned a second stage nerve grafting with sural nerve within four weeks of the primary surgery. The patient was under follow up at the time of reporting (5).

However, not all surgical resections of brachial plexus schwannomas are associated with post-operative neurological deficits. Lee HJ et al analysed the surgical outcome in 19 cases of brachial plexus schwannomas and concluded that these are potentially curable lesion with an acceptable surgical risk of injury to neurovascular structures which can be reduced with precise surgical techniques (6).

Yuce et al further analysed 11 patients of brachial plexus schwannoma which were small in size (<3 cm) but were safely and completely resected using ultrasound-guided microsurgical excision techniques without any peri- or post-operative complications in any of the 11 patients (7).

Kohyama S et al on the other hand described a patient who presented with a left supraclavicular swelling and left sided muscle weakness. Magnetic resonance imaging showed a continuous, multinodular, plexiform tumour from the left C5 to C7 nerve root along the course of the brachial plexus to the left brachia. Intraoperatively, an approximately 40 cm in length tumour, was seen causing extreme enlargement of median and musculocutaneous nerves. Both the nerves showed no response to electric stimulation. They resected the tumour together with a part of the musculocutaneous nerve for biopsy and performed latissimus dorsi muscle transposition in order to repair elbow flexion. Hence, even if during tumour resection a part of the nerve is resected or sustains injury, primary nerve repair using nerve graft or muscle transposition as used in this case, can help to prevent or reduce post- operative neurological sequelae (8).

Before taking up our patient for surgical resection, a detailed counselling preoperatively regarding the surgery, the possible associated complications and its management was done. Under general anaesthesia, we performed complete surgical resection of the tumour with preservation of the nerve fibres. The patient however, developed postoperative stiffness, tingling and numbness, possibly due to the stretching of the nerve fibres during the surgical resection of the tumour. As we were certain that no nerve fascicles were injured during the resection of the tumour, we decided to keep the patient under regular follow up. The patient was also prescribed regular physiotherapy with which she has shown dramatic improvement.

This article highlights the point that a patient presenting with a painless swelling in right supraclavicular region which can easily be misdiagnosed as enlarged lymph nodes in clinical practice could turn out to be a rare diagnosis of brachial plexus schwannoma as in our case report. 

This paper will help apprentice otolaryngologist that such a diagnosis should also be kept in mind while dealing with such swellings. Further this article also elaborates that how with proper work up and pre- operative counselling we can deal with such rare tumors in an amicable way. 

Regarding the incidence of the case, we would like to reiterate that schwannoma as an extracranial nerve sheath tumors rarely affects brachial plexus. Due to the rarity of such tumor and due to brachial plexus anatomic complexity, schwannomas in this region present a challenge for surgeons.

## Conclusion

To conclude, brachial plexus schwannomas should be kept as a differential in patients presenting with supraclavicular neck swellings. As these are potentially curable lesions, a detailed history and examination together with FNAC and imaging studies (MRI and CECT) is necessary in establishing a pre-operative diagnosis thereby reducing the chances of mismanagement. Pre-operative counselling regarding the surgery, its complications and the various options regarding management of neurological complications, if any, helps to improve patient compliance. 
